# Autoinducer-2 of *Streptococcus mitis* as a Target Molecule to Inhibit Pathogenic Multi-Species Biofilm Formation *In Vitro* and in an Endotracheal Intubation Rat Model

**DOI:** 10.3389/fmicb.2016.00088

**Published:** 2016-02-08

**Authors:** Zhengli Wang, Qingqing Xiang, Ting Yang, Luquan Li, Jingli Yang, Hongong Li, Yu He, Yunhui Zhang, Qi Lu, Jialin Yu

**Affiliations:** ^1^Department of Neonatology, Children’s Hospital of Chongqing Medical UniversityChongqing, China; ^2^Ministry of Education Key Laboratory of Child Development and Disorders – Chongqing Key Laboratory of Pediatrics – China International Science and Technology Cooperation Base of Child Development and Critical DisordersChongqing, China

**Keywords:** AI-2, biofilms, PAO1, *Streptococcus mitis*, ventilator-associated pneumonia

## Abstract

*Streptococcus mitis* (*S. mitis*) and *Pseudomonas aeruginosa* (*P. aeruginosa*) are typically found in the upper respiratory tract of infants. We previously found that *P. aeruginosa* and *S. mitis* were two of the most common bacteria in biofilms on newborns’ endotracheal tubes (ETTs) and in their sputa and that *S. mitis* was able to produce autoinducer-2 (AI-2), whereas *P. aeruginosa* was not. Recently, we also found that exogenous AI-2 and *S. mitis* could influence the behaviors of *P. aeruginosa*. We hypothesized that *S. mitis* contributes to this interspecies interaction and that inhibition of AI-2 could result in inhibition of these effects. To test this hypothesis, we selected PAO1 as a representative model strain of *P. aeruginosa* and evaluated the effect of *S. mitis* as well as an AI-2 analog (D-ribose) on mono- and co-culture biofilms in both *in vitro* and *in vivo* models. In this context, *S. mitis* promoted PAO1 biofilm formation and pathogenicity. Dual-species (PAO1 and *S. mitis*) biofilms exhibited higher expression of quorum sensing genes than single-species (PAO1) biofilms did. Additionally, ETTs covered in dual-species biofilms increased the mortality rate and aggravated lung infection compared with ETTs covered in mono-species biofilms in an endotracheal intubation rat model, all of which was inhibited by D-ribose. Our results demonstrated that *S. mitis* AI-2 plays an important role in interspecies interactions with PAO1 and may be a target for inhibition of biofilm formation and infection in ventilator-associated pneumonia.

## Introduction

Interactions among diverse microbial species are dynamic and most likely propel many adaptations, such as biofilm formation in host respiratory tracts and multiple marine habitats (DeLong, 2009; Conway et al., 2012). These interspecies interactions involve the action of multiple genetic and metabolic pathways, which can result in mutualistic or antagonistic effects on bacteria (Xie et al., 2008; Rogers et al., 2010; Conway et al., 2012). It is becoming clear that the different bacteria that coexist in lesions mutually interact and contribute to the pathogenesis of disease (Wagner and Iglewski, 2008; Hibbing et al., 2010; Kim et al., 2010; Baldan et al., 2014). In particular, when different microbes within a community interact with each other, the resulting pathogenesis differs from that of infections caused by the individual component species. In such a context that involves a complex polymicrobial community, single-species microbial analyses are inadequate, not providing much insight into the ecological dynamics of natural habitats (Wagner and Iglewski, 2008; Hibbing et al., 2010; Rogers et al., 2010).

Ventilator-associated pneumonia (VAP) is one of the most common acquired infections in intensive care units (ICUs), occurring in 10–25% of mechanically ventilated patients (Collard et al., 2003). The presence of an endotracheal tube (ETT) is an independent risk factor for developing VAP (Yang et al., 2011; Gil-Perotin et al., 2012). During use, these ETTs accumulate biofilms, which can harbor potentially pathogenic microorganisms, putting bacteria in close proximity to the nose, the pharynx and the surrounding environment (Cairns et al., 2011). Our group previously found that *Streptococcus mitis* (*S. mitis*) and *Pseudomonas aeruginosa* (*P. aeruginosa*) were two of the most common bacteria in biofilms on newborns’ ETTs and in their sputa (Lu et al., 2014; Li et al., 2015b). Recently, we also found that exogenous autoinducer-2 (AI-2) and *S. mitis* could influence the behaviors of *P. aeruginosa* (Li et al., 2015a; Song et al., 2015).

*Streptococcus mitis* is one of the predominant bacteria that colonize the upper respiratory tract of newborns and is generally considered to be harmless (Könönen et al., 2002), and *P. aeruginosa* is one of the microorganisms most frequently responsible for VAP (Safdar et al., 2005; Foglia et al., 2007). It is known that *Streptococcus* is able to produce AI-2, which is encoded by the LuxS gene, but that *P. aeruginosa* is not (Rickard et al., 2006; Williams and Camara, 2009). *P. aeruginosa* infections are difficult to eradicate due to various virulence factors, such as high levels of antibiotic resistance, extracellular proteases and toxins, and the formation of biofilms (Driscoll et al., 2007). These virulence factors are related to quorum sensing (QS), a cell–cell communication mechanism that has been discovered in many species of bacteria (De Kievit, 2009; Gupta et al., 2011; Popat et al., 2012). QS inhibitors (QSIs) are being investigated as antimicrobials because of their potential to reduce symptoms of infectious disease. In particular, AI-2 analogs, such as the C1-alkyl AI-2 analog, isobutyl-DPD (Roy et al., 2013), furanone compound, and D-ribose (Christiaen et al., 2014), have been shown to inhibit QS responses among many bacteria. Unfortunately, however, many contemporary studies on *P. aeruginosa* use mono-species cultures; such experiments overlook the effects of the mutual interactions between coexisting bacteria (De Kievit, 2009).

In the present study, we aimed to explore the effects of *S. mitis* as well as an AI-2 analog (D-ribose) on mono- and co-culture biofilms *in vitro* and in an endotracheal intubation rat model. We focused on the influence of *S. mitis* and D-ribose on PAO1 and specifically evaluated the effects on biofilm formation, virulence and the expression of PAO1 QS genes. This work may provide important insight into VAP caused by multibacterial infection, and particularly neonatal VAP.

## Materials and Methods

### Organisms and Growth Conditions

The species used in the present study were as follows: *S. mitis* (ATCC 49456) and PAO1. The PAO1 was kindly provided by Mr. Zhijin Chen (Third Military Medical University, Chongqing, China; Le et al., 2013). The bacteria were cultured on Columbia sheep blood agar (Pangtong, Chongqing, China) or grown in brain–heart infusion (BHI) broth (Rishui, Qingdao, China). The cultures were incubated at 37°C in a 5% CO_2_, humidified atmosphere. The PAO1 strain was specifically grown overnight on an orbital shaker at 200 rpm. Both bacterial suspensions were standardized to a density equivalent of approximately *A_600_* = 0.5 and then diluted to a working concentration of *A_600_* = 0.05. Additionally, *Vibrio harveyi* BB170 was obtained from Prof. Baolin Sun (University of Science and Technology of China; Zhao et al., 2010). This organism was grown overnight with aeration in AB medium at 30°C (Widmer et al., 2007).

### Growth Assays

PAO1 and *S. mitis* growth in the presence of 50 mM D-ribose (Sigma–Aldrich) was measured at 600 nm at intervals of 2 h for up to 24 h using a spectrophotometer (UV-1800, Shimadzu, Tokyo, Japan). All experiments were performed three times independently (Li et al., 2015a).

### Tested Supernatants and Cells

Supernatants of *S. mitis* were collected at intervals of 2 h for up to 24 h. *V. harveyi* BB170 was also grown overnight. After incubation, the cultures were centrifuged at 12000 rpm at 4°C for 15 min. Cell-free supernatants and cell pellets were then separated using a 0.22 μm Millex filter and used in various experiments.

### AI-2 Assay

Testing for AI-2 was previously described (McNab et al., 2003). Briefly, an overnight culture of *V. harveyi* BB170 was diluted 1:5000 in 2216E medium (Rishui, Qingdao, China), and 20 μl of cell-free culture supernatant or synthesized AI-2 was added to 180 μl of diluted *V. harveyi* cells. Cell-free culture medium from *V. harveyi* BB170 was included as a positive control, and sterile BHI medium and PBS were included as negative controls. The reaction was carried out at 30°C, and light production was monitored using a Thermo Scientific Fluoroskan Ascent FL (Thermo, USA).

### Interactions Between PAO1 and *S. mitis* (Mowat et al., 2010)

To study the direct interactions between the species, standardized suspensions (*A_600_* = 0.05) of PAO1 (50 μl, added to the lower compartment) and *S. mitis* (150 μl, added to the upper compartment) were combined in a 96-well transwell microtiter plate (Corning). BHI broth (150 μl) containing PAO1 (50 μl), BHI broth (50 μl) containing *S. mitis* (150 μl), and BHI broth (200 μl) alone served as three different control groups. We previously determined that the optimal ratio of the bacterial species is 1:3 (PAO1:*S. mitis*) (data not shown). Then, 22 μl of D-ribose (500 μM) or sterilized ddH_2_O was added to each well. The plates were subsequently incubated at 37°C in a 5% CO_2_, humidified atmosphere for 24 h.

To study the indirect interactions between the species, we employed co-cultivation of PAO1 with supernatant from *S. mitis* or with methanol-treated *S. mitis* cells. The effect of the killed bacterial cells on PAO1 biofilm formation was investigated as described previously (Mowat et al., 2010). Briefly, PAO1 and *S. mitis* were centrifuged, washed twice in phosphate-buffered saline (PBS) and resuspended in 100% methanol for 2 h. Dead cells were then centrifuged and washed three times with PBS to remove any remaining trace of methanol. Finally, the dead cells were resuspended to *A_600_* = 0.05. To confirm bacterial killing, aliquots of the bacterial cells were spread onto blood agar plates and incubated overnight at 37°C and 5% CO_2_. A total of 100 μl of the standardized suspension of PAO1 was combined with methanol-killed bacterial cells (PAO1 or *S. mitis*) in a 96-well microtiter plate and incubated for 24 h at 37°C and 5% CO_2_. The biomass was then quantified by crystal violet assay.

### Quantification of Biofilm Biomass by Crystal Violet Assay

The biofilm biomass was assessed using a modified version of a protocol first developed by Christensen et al. (1985) and subsequently modified by O’Toole and Kolter (1998). At each time point, the spent culture medium was removed from each well, and the adherent cells were washed with PBS. These cells were then air dried, after which 0.1% (w/v) crystal violet solution was added for 5 min. After washing, the crystal violet-stained biofilms were also air dried. To quantify the biofilm biomass, the crystal violet was removed by adding 200 μl of 95% ethanol to each well, and the *A*_570_ of the solubilized dye was obtained using an Epoch microplate spectrophotometer (BioTek).

### Transwell Biofilm Assay

For transwell studies, uncoated 24-well transwell polystyrene cell culture plates (Corning) with one coverslip per well were inoculated with 0.25 ml (*A*_600_ = 0.05) of PAO1 in the lower compartment and 0.75 ml of BHI broth or *S. mitis* (*A*_600_ = 0.05) in the upper compartment. Half of the PAO1&*S. mitis* wells also received D-ribose at a final concentration of 50 mM. After 24 h of incubation at 37°C and 5% CO_2_, the liquid medium was removed. The coverslips were then gently washed with PBS, and the biofilm mass, cell number, and structure were analyzed using microscopic techniques (see below).

### Biofilm Structure and Viability Counts

For scanning electron microscopy (SEM) studies, an inoculum was prepared as previously described (Li et al., 2008). For confocal laser scanning microscopy (CLSM) studies, the biofilms were stained with SYTO 9-propidium iodide LIVE/DEAD BacLight (Invitrogen, USA) according to the manufacturer’s protocol. After staining, the biofilms were observed using a CLSM system (Radiance 2000; Bio-Rad, Hemel Hempstead, UK) consisting of a microscope (Nikon, Tokyo, Japan) and a krypton-argon mixed-gas laser source, with an argon laser with a 488-nm excitation wavelength and a helium/neon laser with a 546-nm excitation wavelength. Three-dimensional images were obtained using NIS-Elements Viewer software (Nikon, Japan). Stacks of horizontal-plane images captured by CLSM were subjected to quantitative image analysis using COMSTAT software (Heydorn et al., 2000a,b); this program calculates several characteristic biofilm parameters for each image stack, such as the total biomass, the maximum thickness, the average thickness, the roughness coefficient, and the surface area of the biomass.

In parallel experiments, samples were prepared for RNA extraction and viability counts. Viable cell numbers were obtained from biofilms on coverslips in 24-well polystyrene cell culture plates after two gentle washes with PBS. The resulting suspensions were serially diluted in PBS, and 50 μl aliquots were plated on Columbia sheep blood agar. Colony-forming units (CFU) were counted after 16–18 h of incubation, and the distinct colony morphologies allowed for differentiation between the two species.

### RNA Fixation, Extraction, and Reverse Transcription

Total RNA was extracted from the bacteria adhering to the coverslips using the TaKaRa MiniBEST Universal RNA Extraction Kit (TaKaRa Bio). Reverse transcription was then performed using the TaKaRa PrimeScript RT Reagent Kit with gDNA Eraser (TaKaRa Bio) according to the manufacturer’s instructions. cDNA samples from the same biological sample were pooled, diluted six times and stored at –20°C until use.

### Quantitative Real-Time Polymerase Chain Reaction (Q-PCR) Analysis

SsoFast EvaGreen Supermix (Bio-Rad) was used for real-time amplification and for visualization of the amplified cDNA. In particular, 1 μl of a cDNA dilution, 0.4 μl each of forward and reverse primers, 5 μl of SsoFast EvaGreen Supermix, and 3.2 μl of sterile H_2_O were added to the reaction wells. A no-template control (NTC) was included during each Q-PCR experiment to check the purity of the reagents. Each reaction was performed in duplicate, and the experiments were repeated four times, with different RNA samples used each time.

The Q-PCR reactions were performed in a CFX96 Real-Time PCR Detection System (Bio-Rad). The thermal cycling conditions started with 95°C for 30 s, followed by 40 cycles of 95°C for 5 s and 60°C for 5 s. The primer sets used for these analyses are listed in **Table 1**. A single sharp peak and a single band of the expected size were observed in the melting curve and in the agarose gel, respectively. The identities of the products were confirmed by DNA sequencing.

**Table 1:** Sequences of gene-specific primers used for quantitative real-time polymerase chain reaction (Q-PCR).

Target gene	Primer sequence	Category	Function
*lasI-F*	GGCTGGGACGTTAGTGTCAT	QS	Synthase 3-oxo-C12-HSL
*lasI-R*	AAAACCTGGGCTTCAGGAGT		
*lasR-F*	ACGCTCAAGTGGAAAATTGG	QS	Receptor 3-oxo-C12-HSL
*lasR-R*	TCGTAGTCCTGGCTGTCCTT		
*rhlI-F*	AAGGACGTCTTCGCCTACCT	QS	C4-HSL synthase
*rhlI-R*	GCAGGCTGGACCAGAATATC		
*rhlR-F*	CATCCGATGCTGATGTCCAACC	QS	C4-HSL receptor
*rhlR-R*	ATGATGGCGATTTCCCCGGAAC		
*rpsL-F*	GCAACTATCAACCAGCTGGTG	Normalization gene	30S ribosomal protein S12
*rpsL-R*	GCTGTGCTCTTGCAGGTTGTG		

*QS, quorum sensing.*

### Processing of Q-PCR Results

Whether the expression of certain target genes in PAO1 could be reproducibly induced or affected by systematic co-culturing under multi-strain growth conditions was investigated. Relative mRNA expression was quantified using the efficiency-corrected comparative *C_t_* method, and the expression levels of the same genes in mono- and co-cultures of PAO1 and *S. mitis* were compared. In this study, the expression of target genes in mono-cultures acted as the control. The *C_t_* values of both the control and the genes in question were then normalized to that of the PAO1 housekeeping gene (*rpsL*) (Yang et al., 2012).

After Q-PCR amplification, the comparative threshold method (*ΔΔC_t_* analysis) was applied to evaluate the relative changes in gene expression in the Q-PCR experiments. The computer programs GenEx (Bio-Rad) and Excel (Microsoft) were used to solve the following equation: ΔΔCt = ΔΔCt, _sample_–ΔΔCt,_reference_ (Bio-Rad) (Conway et al., 2012).

### Endotracheal Intubation Rat Model

To assess how lung infection changes between ETTs covered in single- and dual-species biofilms, an endotracheal intubation rat model was used. For this purpose, disposable sterile plastic scalp acupuncture tubes of 3.0 mm in diameter were cut to 1 cm in length and precoated with bacteria before endotracheal intubation. The prepared tubes were specifically immersed in the same bacterial suspension as that used in the transwell biofilm assay described above. After biofilms formed on the surface of the inoculation tubes, to estimate the bacterial count in these biofilms, the bacteria were detached from the tubes by consecutive ultrasonic shaking and vortexing for 2 min and 5 min, respectively. Our preliminary results indicated that this process did not affect cell culturability. To make the density of bacteria in each group approximately equivalent, we washed the tubes covered in both PAO1 and *S. mitis* four times, whereas the other tubes were washed 3 times. These different protocols were used because the mixed PAO1&*S. mitis* biofilm contained more bacteria than the PAO1 biofilm did. The CFU value of the PAO1 biofilm and the mixed PAO1&*S. mitis* biofilm after 72 h of incubation and before endotracheal intubation was approximately 1 × 10^7^, whereas the CFU value of the *S. mitis* biofilm was less than ∼10^4^.

Sixty adult female Sprague–Dawley (SD) rats, each weighing 200–220 g, were randomly allocated to 4 groups with 15 rats each. Intubations with PAO1 (P group), PAO1&*S. mitis* (PS group), PAO1&*S. mitis*&D-ribose (PSD group), and *S. mitis* (S group) biofilm-covered tubes were chosen as the four infection groups, and intubation with sterile tubes served as the control. The PSD group was treated with ultrasonic atomization-based inhalation of D-ribose (500 mM, 5 ml) for 30 min every day, whereas the other groups were treated with ultrasonic atomization-based inhalation of PBS.

The animals were purchased from the Chongqing Medical University Laboratory Animals Center (Chongqing, China). All rats were housed in a pathogen-free environment and received sterile food and water in the Laboratory Animal Center at the Children’s Hospital of Chongqing Medical University. The procedure of endotracheal intubation was previously described (Li et al., 2011). Animal studies were conducted according to protocols approved by the Chongqing Medical University (Chongqing, China) Institutional Animal Care and Use Committee.

### Histological and Bacteriological Observation

Half of each left lung was homogenized and prepared for bacterial counting, as described previously (Li et al., 2011). The remaining parts of the lungs from all rats in all groups were fixed in 10% formalin buffer and subjected to histopathological examination. This lung histological examination was performed as described previously (Ding et al., 2014). Briefly, at 7 days post-intubation, the rats were euthanized, and their left lungs were ligated and aseptically excised and homogenized. The homogenates were then serially diluted and plated onto Columbia sheep blood agar plates, which were incubated overnight at 37°C and 5% CO_2_. The colonies on the plates were subsequently counted to estimate CFU values. Simultaneously, the right lung was lavaged with 3 ml of PBS; this fluid was instilled and withdrawn three times. Following the lavage, the total cell count of the bronchoalveolar lavage fluid (BALF) was measured using a cell counter (Countstar, Beijing, China). The protein levels of IL-4, IL-10, and IL-6 were also measured using commercially available ELISA kits (Beijing 4A Biotech Co., Ltd., Beijing, China) according to the manufacturer’s instructions. The BALF samples were assayed in duplicate.

### Statistical Analysis

All experiments were performed in triplicate unless stated otherwise. Statistical analyses were performed using SPSS 19 software. The data are presented as the mean ± SD. The *t*-statistic was used to determine other significant differences between two groups, and a *P*-value of <0.05 was considered statistically significant. Moreover, comparisons of mortality from infection were performed using Fisher’s exact test.

## Results

### Effects of D-Ribose on *P. aeruginosa* Growth

We first investigated the effect of D-ribose on planktonic bacterial growth. The 50 mM concentration of D-ribose did not influence the growth of the planktonic PAO1 cultures, whereas it induced the growth of *S. mitis* in the stationary phase (**Figure 2A**).

### Bioluminescence Assay

Cell-free supernatants harvested from representative strains of *S. mitis*, PAO1 and BB170 after 14 h of growth were screened for AI-2-like activities. AI-2 was observed in the S group and BB170 group, whereas it was not observed in the P group (**Figure 2B**).

### Mono- and Dual-Species Biofilm Formation

**FIGURE 1:**
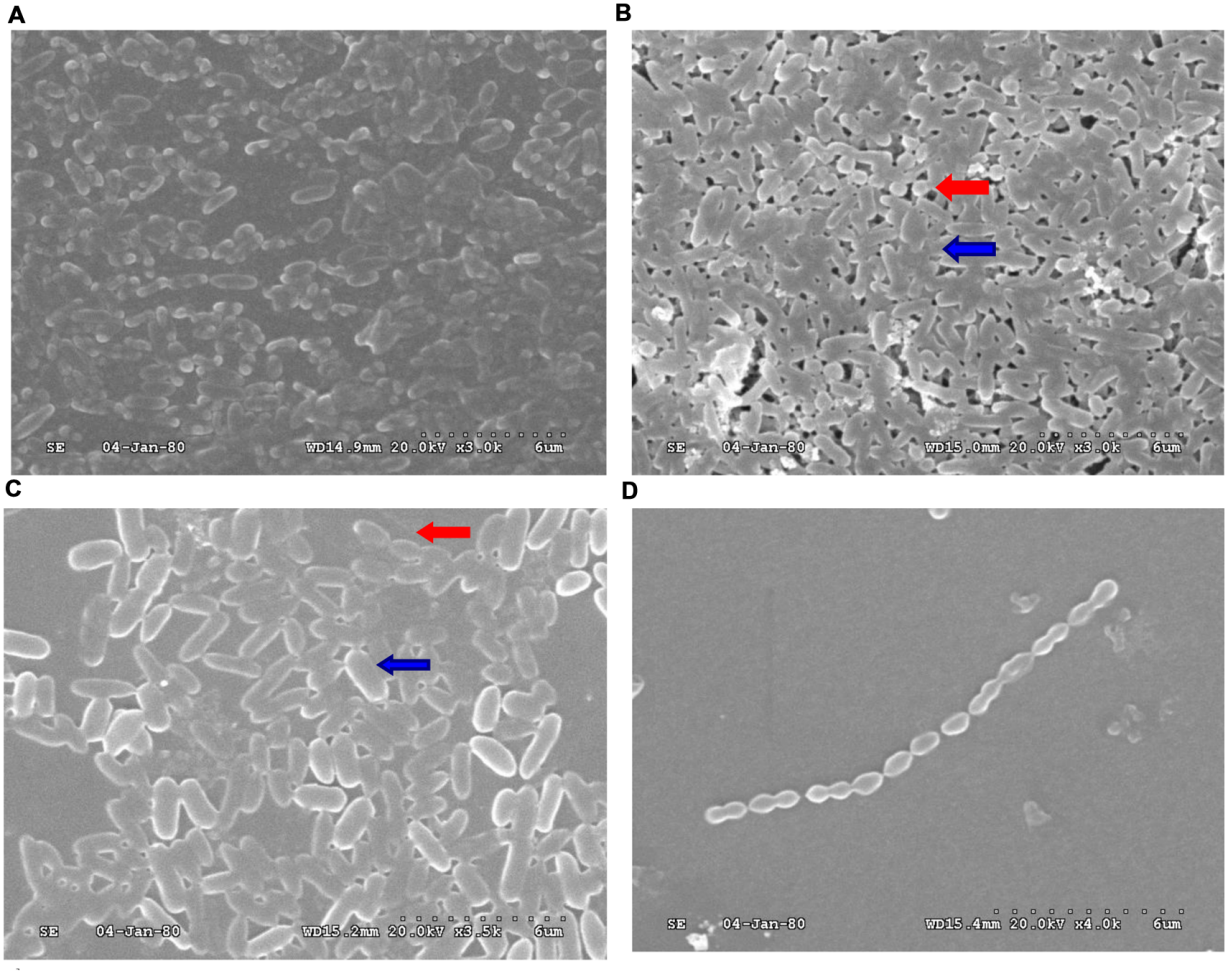
**Scanning electron microscopy (SEM) analysis of biofilms obtained from PAO1 and *Streptococcus mitis*. (A)** PAO1 mono-culture. **(B)** PAO1 and *S. mitis* co-culture. **(C)** PAO1 and *S. mitis* co-culture with 50 mM D-ribose. **(D)** *S. mitis* mono-culture. Red arrows: *S. mitis*; Blue arrows: PAO1.

**FIGURE 2:**
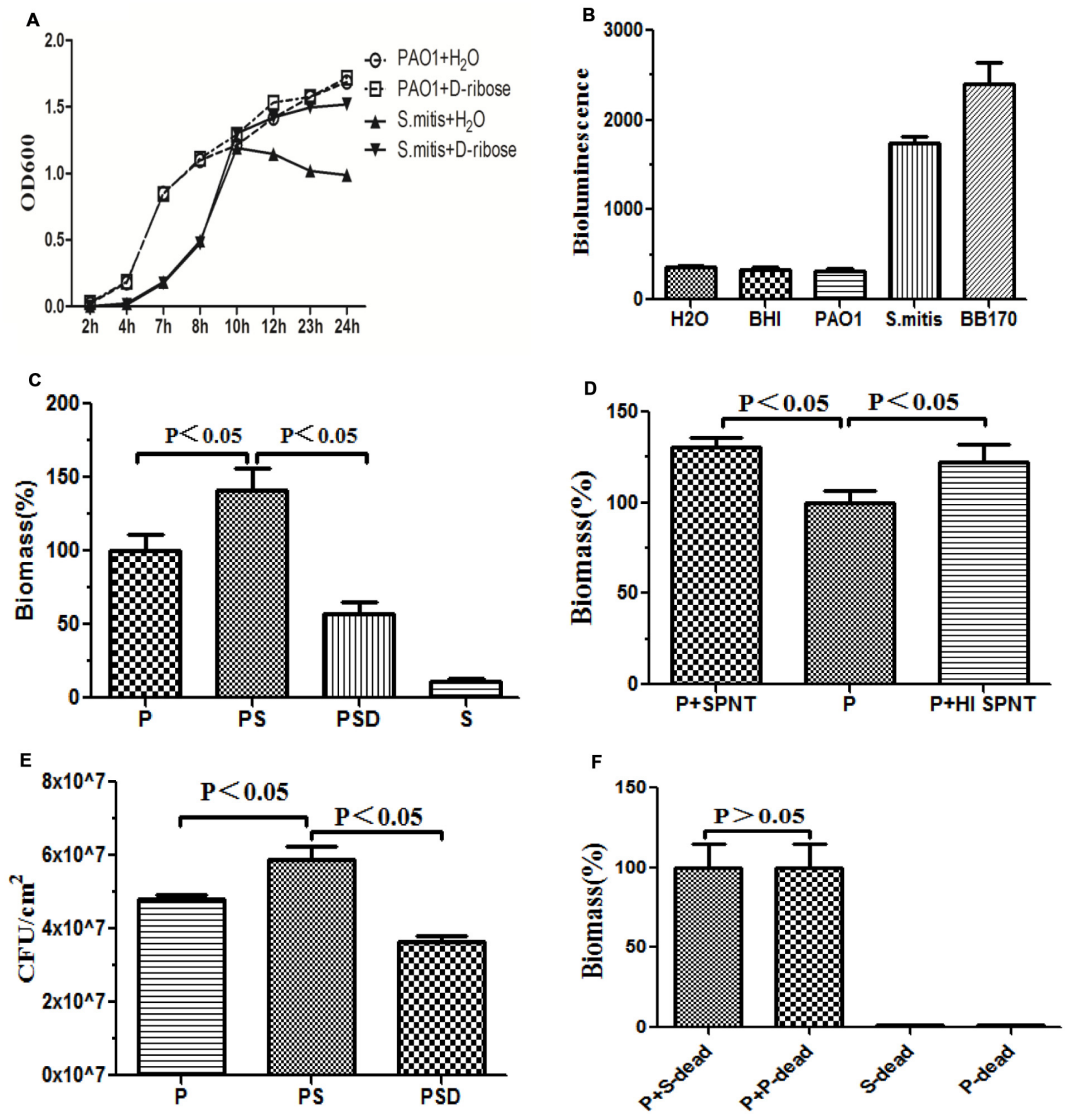
**Quantification of biofilm formation by crystal violet staining.** Quantification of the biofilm biomass by spectrophotometry (OD 570). The error bars indicate standard deviations. P represents PAO1 group; PS represents PAO1&*S. mitis* group; PSD represents PAO1&*S. mitis* group co-culture with D-ribose (50 mM). **(A)** Effects of D-ribose on planktonic growth of PAO1 and *S. mitis*. Cells were grown in BHI medium, in the presence of 50 mM concentrations of D-ribose or dH_2_O. The data represent mean values of three independent experiments. **(B)** The biomass of co-cultured PAO1 and *S. mitis* was significantly higher than that of co-cultured PAO1 and *S. mitis* with 50 mM D-ribose and mono-cultured PAO1 or *S. mitis*. **(C)** PAO1 was exposed to fresh spent supernatant (SPNT) or heat-treated supernatant (HI SPNT) from *S. mitis*. Both the SPNT and the HI SPNT from *S. mitis* could increase the biomass of the PAO1 biofilm. **(D)** Bacterial colony counts of mono- and dual-species biofilms. **(E)** No significant difference was found between co-cultures of PAO1 with dead *S. mitis* cells and co-cultures with dead PAO1 cells. ‘P-dead’ represents dead PAO1 cells, and ‘S-dead’ represents dead *S. mitis* cells. **(F)** Bioluminescencing activity of partially purified *S. mitis* AI-2 and BB170 (AB medium for *V. harveyi*, BHI for *S. mitis* and PAO1).

To confirm whether the reciprocal interactions between the two species could affect their capacity to produce biofilms, we first investigated mono-species biofilms formed by PAO1 or *S. mitis*. As shown in **Figure 1**, the PAO1 strain formed flat, tight biofilms with little heterogeneity and few viable cells, whereas *S. mitis* alone did not form biofilms. When the two strains were co-cultured (**Figure 1B**), the biofilms had a denser, thicker structure. Based on analysis using COMSTAT software, PAO1&*S. mitis* biofilms showed significant structural differences compared with the P group and the PSD group. As is shown in **Table 3**, the biomass, the average thickness, the maximum thickness and the surface area of the biomass all increased in the PS group, whereas the roughness coefficient showed the opposite trend. Interestingly, D-ribose (50 mM) significantly inhibited co-culture biofilm formation (**Table 3**), whereas there was no significant effect on PAO1 viability or biofilm formation (**Figure 2A**).

Under co-culture conditions, we noticed that the biomass and the total number of live cells within the biofilms increased. Crystal violet assays revealed that *S. mitis* could not form biofilms (**Figures 1D** and **2C**). Co-cultivation of PAO1 with *S. mitis* resulted in an increase in the total biomass by 41.18% compared with PAO1 mono-species culture, whereas adding D-ribose showed the opposite trend (**Figure 2C**). The addition of ethanol-treated *S. mitis* cells did not have this effect on PAO1 cultures (**Figure 2F**). The total bacterial colony counts for the PS group also increased by 28.78% compared with the counts in PAO1 biofilms (**Figure 2E**). In addition, *S. mitis* accounted for approximately 8% of the dual-species biofilms (data not shown). Exposure to *S. mitis* supernatant also triggered PAO1 adhesion, increasing the biofilm biomass by 30.77%. Heat-treating the supernatant did not significantly reduce this effect, as the biofilm biomass still increased by 22.25% (**Figure 2D**).

### Gene Expression Analyses of Mono- and Co-Cultures by Q-PCR

**Table 2** presents the normalized results as relative gene expression under the co-culture conditions compared with expression under the mono-culture conditions. Significant differences were observed in the relative levels of the target genes (*lasR, lasI, rhlR*, and *rhlI* genes), with significant overexpression under co-culture conditions.

**Table 2:** Gene expression in PAO1&*S. mitis* co-cultured biofilms in relation to that in PAO1 mono-cultured biofilms.

Gene	Fold change
*lasI*	2.80 ± 0.64
*lasR*	3.25 ± 2.01
*rhlI*	2.08 ± 0.49
*rhlR*	2.04 ± 0.61

*The data represent the mean fold changes in gene expression between co-cultured biofilms and mono-cultured biofilms and were analyzed from four biological repeats.*

### Pathogenicity in the VAP Model

To determine whether ETTs covered with mixed PAO1&*S. mitis* biofilms could increase the mortality rate and aggravate lung infection and to analyze the effects of D-ribose, we measured the mortality rate, performed bacteriological examinations and measured the total cell count in the BALF in all groups.

#### Survival

**FIGURE 3:**
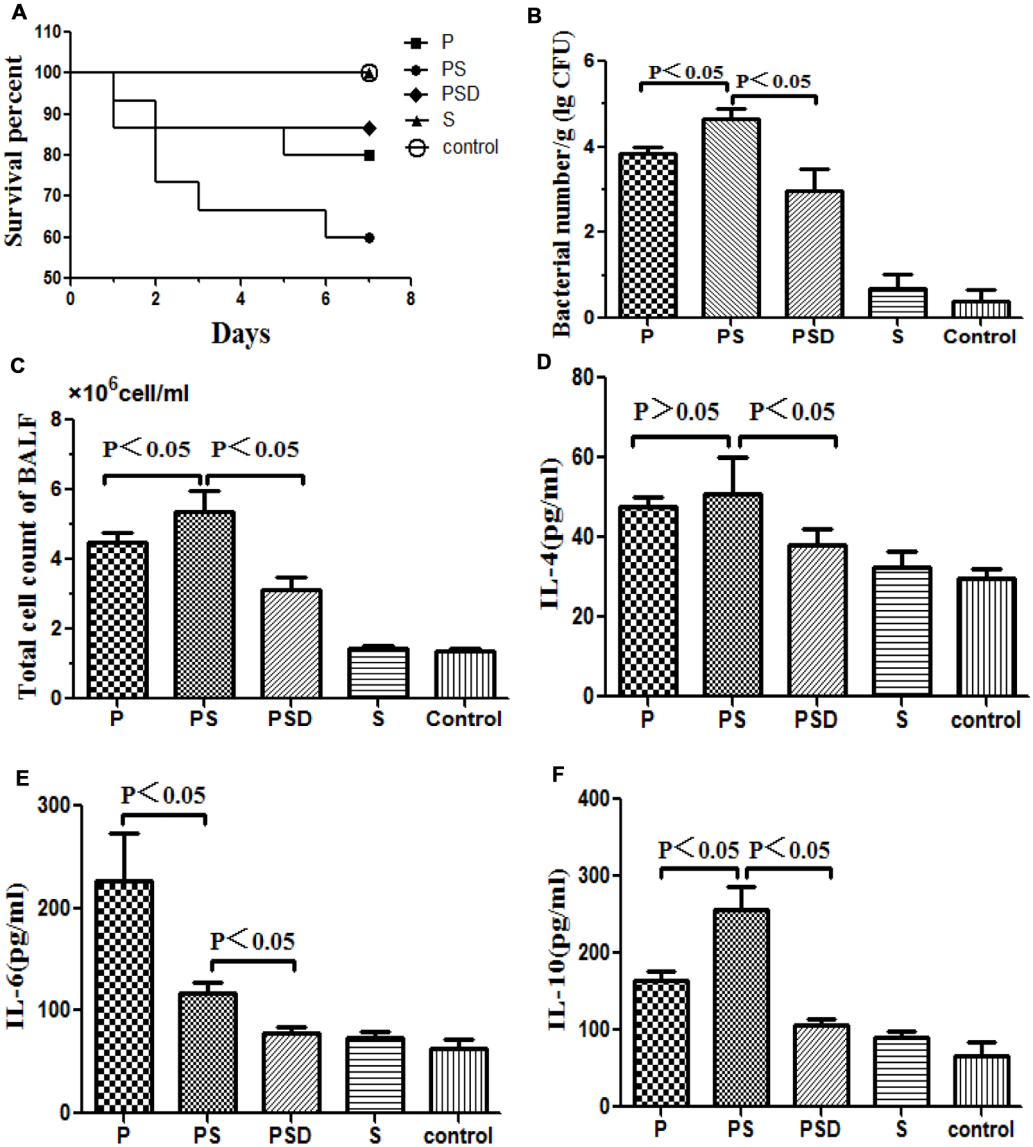
**Survival rates of rats inoculated with different biofilm-covered ETTs.** Fifteen rats in each group were inoculated with PAO1 (**P**), PAO1&*S. mitis* (**PS**), or *S. mitis* (**S**) biofilm-covered ETTs, and sterilized tubes were used as controls. The **PSD** represents PS group was treated with D-ribose, whereas the other four groups were treated with PBS. **(A)** The survival rate was estimated at the indicated times, and the results are displayed as a Kaplan–Meier plot. The survival time of the PAO1&*S. mitis* group was significantly shorter than the survival times of the other three groups (*P* < 0.05). **(B)** Number of bacteria in the lung tissue (log CFU per gram). **(C)** Total numbers of cells in the BALF of the different groups. **(D–F)** Protein levels of IL-4, IL-6, and IL-10 in the BALF of the different groups.

As shown in **Figure 3A**, the mortality rate over the 7 days following intubation was higher when the tubes were covered in mixed PAO1&*S*. *mitis* biofilms compared with when the tubes were covered in PAO1 biofilms, and D-ribose reduced this effect. However, the difference was not statistically significant (*P* > 0.05), which may have been due to the small sample size.

#### Histological and Bacteriological Examination

**FIGURE 4:**
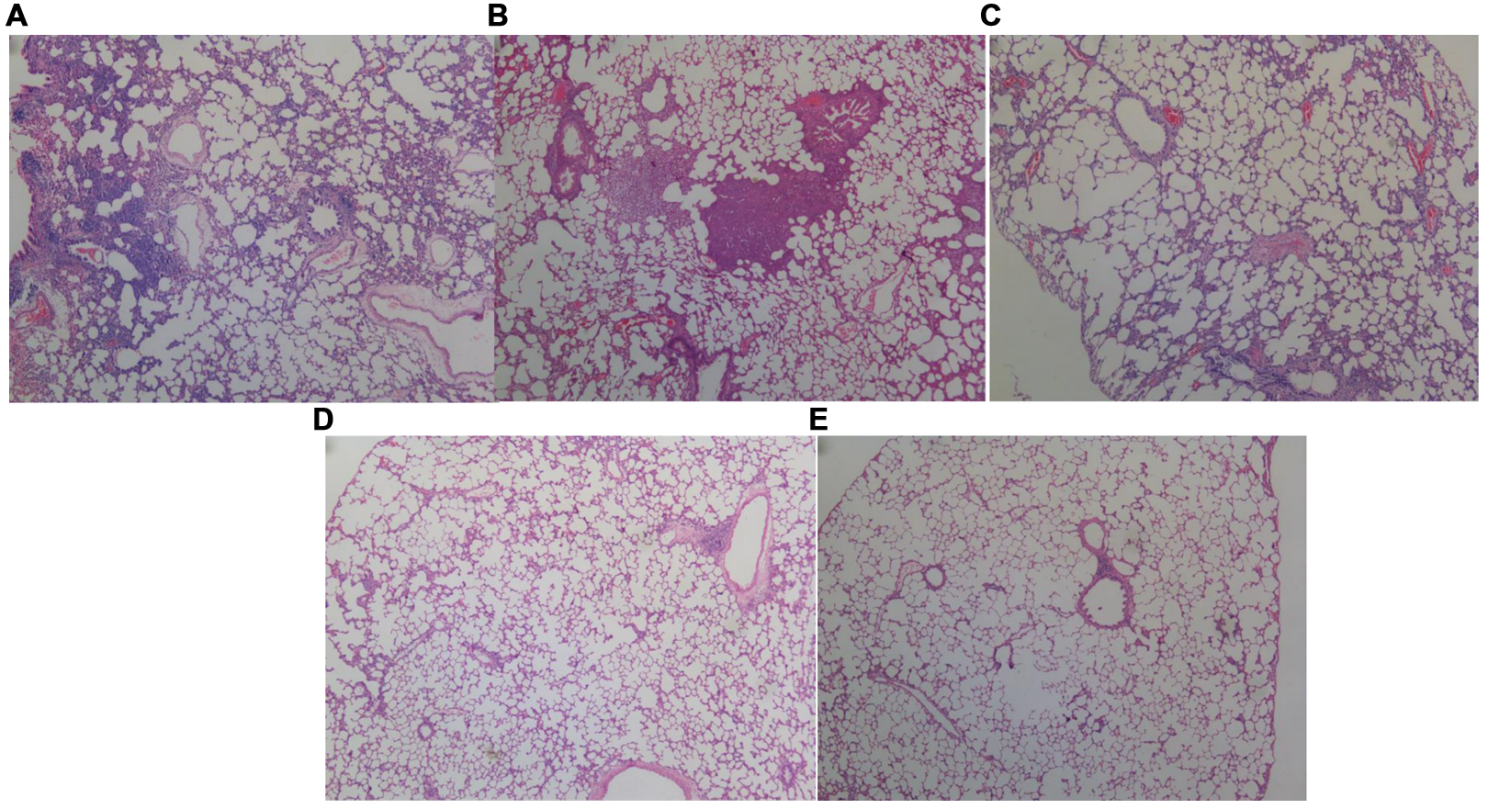
**Histopathological analysis of the lungs of rats inoculated with different biofilm-covered ETTs.** Sections of lungs stained with hematoxylin-eosin on day 7 post-infection are shown. **(A–E)** Depict the lungs of P group, PS group, PSD group, S group, and the control group, respectively. The destination area was selected using an inverted microscope at 400×. Both **(A,B)** show large numbers of inflammatory cells infiltrating the alveolar spaces and lung necrosis. Furthermore, the pulmonary tissues in the PAO1&*S. mitis* group presented significantly more inflammation than those in the PAO1 group.

Respiratory infection, which was measured based on histological examination and CFU per gram, occurred in all inoculated mice. As shown in **Figures 3** and **4**, the PS group exhibited a heavier bacterial burden and more serious pathological changes, and treatment with D-ribose reduced this effect. In contrast, the bacterial cell counts in the lungs of both the control group and the S group were much lower or negative (*P* < 0.05).

#### Total Cell Count and Cytokine Protein Levels in the BALF

As shown in **Figures 3D–F**, the total number of cells and the IL-10 level in the BALF were significantly higher in the PS group than in the other three groups, and the expression of IL-10 decreased following treatment with D-ribose (*P* < 0.05). However, there was no significant difference in IL-4 between the PS group and the P group. In contrast, the IL-6 level was significantly lower in the PS group than in the P group.

## Discussion

Biofilms on the surface of neonatal ETTs represent a unique environment affected by complex microbial ecology. However, to date, relatively little is known about the interactions within biofilms, even though an understanding of these interactions is very important for the development of treatments for biofilm-related infectious diseases. Our group recently found that *S. mitis* and *P. aeruginosa* were two of the most common bacteria in biofilms on newborns’ ETTs and in their sputa (Lu et al., 2014; Li et al., 2015b). *P. aeruginosa*, which is one of the major pathogens in VAP, is hard to treat due to its formation of biofilms, whereas *S. mitis* is thought to be part of the normal flora in the neonatal nasopharynx (Könönen et al., 2002). Recently, we also found that exogenous AI-2 and *S. mitis* could influence the behaviors of *P. aeruginosa* (Li et al., 2015a; Song et al., 2015). As AI-2 is a universal signal for interspecies communication (Sun et al., 2004), which is required for multi-species biofilm growth (Shao et al., 2007), interruption of AI-2 signaling is a potential way to inhibit biofilms (Jang et al., 2013; Roy et al., 2013). The inhibitory effect of D-ribose on multispecies biofilm formation is likely attributable to competitive binding of D-ribose to the AI-2 receptor (Jang et al., 2013).

Multi-species biofilms often possess combined metabolic activity that is greater than that of the component species (Gilbert et al., 1997; Giaouris et al., 2013). Palmer et al. (2001) demonstrated that the co-aggregating partnership of *S. oralis* and *Actinomyces naeslundii* formed a nutritionally beneficial, mutualistic relationship that allowed each to grow where neither could grow alone. In the present study, we first examined the direct interactions between PAO1 and *S. mitis* in co-culture biofilms. Different patterns were observed in these co-culture biofilms. In particular, mono-cultures of *S. mitis* could not form biofilms, but the bacterium could facilitate PAO1 microcolony formation and adhesion. Mono-cultures of PAO1 also formed loosely packed microcolony structures after 24 h. Denser, thicker structures with more live cells were formed when PAO1 was co-cultured with *S. mitis* (**Figures 1** and **2**; **Table 3**). These results are in agreement with reports that *S. mitis* increases biofilm formation by dental and periodontal pathogens (Schlafer et al., 2012), whereas adding an AI-2 analog (D-ribose) can inhibit dual-species biofilm formation (Jang et al., 2013). These findings indicated that *S. mitis* could promote PAO1 biofilm formation and pathogenicity but that an AI-2 analog (D-ribose) could inhibit these effects.

**Table 3:** COMSTAT analysis of biofilm parameters.

Group^∗∗^	Total biomass (μm^3^/μm^2^)	Maximum thickness (μm)	Average thickness (μm)	Roughness coefficient	Surface area of biomass in image stack (×10^6^)
P	4.76 ± 0.25*	14 ± 2.29	9.34 ± 2.04*	0.44 ± 0.04*	1.96 ± 0.12*
PS	10.42 ± 3.18	16.13 ± 2.57	14.57 ± 2.81	0.11 ± 0.06	2.93 0.46
PSD	1.48 ± 0.93*	7.13 ± 2.97*	2.98 ± 2.00*	1.02 ± 0.55*	0.82 ± 0.53*

*^∗^Statistically significant compared to PS group (*P* < 0.05). ^∗∗^P represents PAO1 group; PS represents PAO1&*S. mitis* group, The PSD represents the PAO1&*S. mitis* group was treated with D-ribose, whereas the other two groups were treated with PBS.*

We have shown that the interaction between PAO1 and *S. mitis* is mediated not only by direct contact but also by secreted extracellular molecules. This concept is in accordance with previous investigations that examined supernatants from bacterial strains found in the respiratory and gastrointestinal tracts and identified *P. aeruginosa* culture supernatants as having inhibitory properties (Mowat et al., 2010). We found that the addition of 0.22 μm-filtered supernatants (from either heat-killed or live cultures) increased the biomass of PAO1 biofilms (**Figure 3D**), indicating that the release of small, heat-stable molecules was responsible for this increase in biomass.

Quorum sensing plays a key role in bacterial adhesion and biofilm formation (Haussler and Becker, 2008; Williams and Camara, 2009). In the current study, we found that *S. mitis* enhanced the expression of the PAO1 QS genes, including *lasI, lasR, rhlI*, and *rhlR* (**Table 2**), and that D-ribose (50 mM) inhibited co-culture biofilm formation. The inhibitory effect of D-ribose on biofilm formation is likely attributable to competitive binding of D-ribose with AI-2 for *RbsB* (Jang et al., 2013). Interestingly, at the concentration used in this study, D-ribose did not affect the viability of PAO1 or *S. mitis*, but it could improve the bacterial density of *S. mitis* in the stationary phase. The *rhl* QS system is one of the major signaling pathways in the development of self-aggregating properties (De Kievit, 2009). Meanwhile, the *las* QS system has been found to be essential for the creation of mature, differentiated biofilms (Davies et al., 1998). QS system inhibition is widely accepted as a promising tool for the treatment of *P. aeruginosa* infections (Xavier and Bassler, 2005; Zhu and Li, 2012). AI-2 is a universal QS molecule that mediates intra- and interspecies communication, including initial bacterial aggregation and the production of virulence factors (Sun et al., 2004). Thus, bacterial QS compounds change the physiology of conspecific members of the population and represent another possible explanation for the changes in gene expression observed during co-culture (Conway et al., 2012).

To further investigate the differential virulence of single- and dual-species biofilms *in vivo*, we used an endotracheal intubation rat model. Animal lung infection models have been established previously (Li et al., 2011). This particular model possesses at least somewhat similar properties as human catheter-associated lung infections observed in clinical settings.

As shown in **Figures 3** and **4**, higher rates of mortality and more serious infections were observed in the PS group than in the other three groups due to a heavier bacterial burden, more serious pathological changes, and more inflammatory cells in the BALF. Similarly, Whiley et al. (2014) reported that the pathogenic potential of the *P. aeruginosa* Liverpool Epidemic Strain (LES) can be enhanced by the presence of oral commensal streptococci, and *S. mitis* has also been shown to potentiate LES virulence factor production in co-culture biofilms (Whiley et al., 2015). Interestingly, in the present study, the concentration of IL-6 in the BALF was lower in the PS group than in the P group, whereas the IL-10 level was significantly higher than in the P group (*P* < 0.05). IL-10 has emerged as a key immunoregulator during infection (Oswald et al., 1992; Sieling et al., 1993). High pathogen loads drive excessive Th1 responses (Anderson et al., 2005), and these in turn promote the development of self-limiting, adaptive, IL-10-producing T cells that dampen the Th1 response (Belkaid et al., 2001; Anderson et al., 2007). These events establish a positive feedback loop whereby T cell-derived IL-10 further inhibits antimicrobial immune responses, allowing inevitably fatal infections to develop (Couper et al., 2008). In addition, IL-10 can synergize with IL-4 to inhibit macrophage cytotoxic activity and limits the production of proinflammatory cytokines (i.e., IL-6), suppressing killing of pathogens (Oswald et al., 1992; Couper et al., 2008). These findings are consistent with our present study, which suggested that *S. mitis* could promote the virulence of PAO1 and induced evasion of immune clearance in the host and that D-ribose could inhibit these effects. We presume that the AI-2 produced by *S. mitis* may play an important role, although other unknown substances cannot be excluded. Therefore, the exact mechanism remains to be revealed.

The concern over rising antibiotic resistance necessitates exploration of alternative approaches in antimicrobial therapy (Ahmed et al., 2007). The widespread nature of AI-2- mediated communication among bacteria renders it one possible therapeutic target (Roy et al., 2010). For future studies, it will therefore be important to clarify the role of AI-2 analogs in counteracting mixed bacterial infections and whether AI-2 analog compounds could serve as lead molecules for drug development.

## Conclusion

The present study demonstrated that (1) *S. mitis* promoted PAO1 biofilm formation, pathogenicity and expression of QS genes; (2) ETTs covered in dual-species biofilms increased the mortality rate and aggravated lung infection compared with ETTs covered in mono-species biofilms in an endotracheal intubation rat model; and (3) all of these effects were inhibited by an AI-2 analog (D-ribose). Overall, our results demonstrated that *S. mitis* AI-2 plays an important role in interspecies interactions with PAO1 and may be a target for inhibition of biofilm formation and infection in VAP.

## Author Contributions

ZW and JYu conceived and designed the study, ZW and QX performed the study, TY, LL, JYa, and HL analyzed and interpreted the data, ZW and YH wrote the manuscript. JYu, YZ, and QL revised the manuscript. All authors read and approved it for publication.

## Conflict of Interest Statement

The authors declare that the research was conducted in the absence of any commercial or financial relationships that could be construed as a potential conflict of interest.
